# Two-Photon Polymerization Printing with High Metal Nanoparticle Loading

**DOI:** 10.1021/acsami.3c10581

**Published:** 2023-10-10

**Authors:** Nuzhet I. Kilic, Giovanni M. Saladino, Sofia Johansson, Rickard Shen, Cacie McDorman, Muhammet S. Toprak, Stefan Johansson

**Affiliations:** † Department of Materials Science and Engineering, Microsystems Technology, Uppsala University, SE 75103 Uppsala, Sweden; ‡ Department of Applied Physics, Biomedical and X-ray Physics, KTH Royal Institute of Technology, SE 10691 Stockholm, Sweden; § Department of Materials Science and Engineering, Biomedical Engineering, Science for Life Laboratory, Uppsala University, SE 75103 Uppsala, Sweden; ∥ Kanthal AB, SE 73427 Hallstahammar, Sweden; ⊥ Alleima Advanced Materials, Palm Coast, Florida 32164, United States

**Keywords:** additive manufacturing, two-photon polymerization, metal nanoparticles, nanoparticle surface engineering, X-ray fluorescence

## Abstract

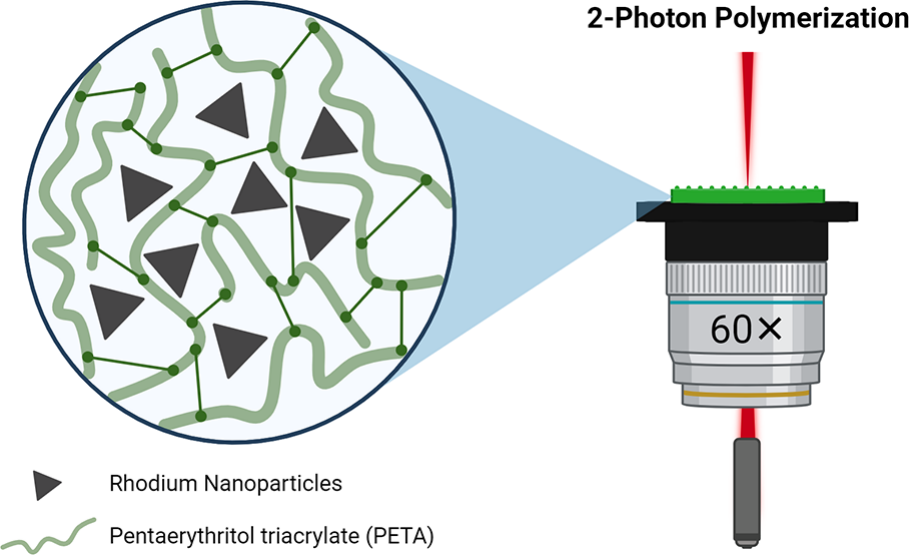

Two-photon polymerization (2PP) is an efficient technique to achieve high-resolution, three-dimensional (3D)-printed complex structures. However, it is restricted to photocurable monomer combinations, thus presenting constraints when aiming at attaining functionally active resist formulations and structures. In this context, metal nanoparticle (NP) integration as an additive can enable functionality and pave the way to more dedicated applications. Challenges lay on the maximum NP concentrations that can be incorporated into photocurable resist formulations due to the laser-triggered interactions, which primarily originate from laser scattering and absorption, as well as the limited dispersibility threshold. In this study, we propose an approach to address these two constraints by integrating metallic Rh NPs formed ex situ, purposely designed for this scope. The absence of surface plasmon resonance (SPR) within the visible and near-infrared spectra, coupled with the limited absorption value measured at the laser operating wavelength (780 nm), significantly limits the laser-induced interactions. Moreover, the dispersibility threshold is increased by engineering the NP surface to be compatible with the photocurable resin, permitting us to achieve concentrations of up to 2 wt %, which, to our knowledge, is significantly higher than the previously reported limit (or threshold) for embedded metal NPs. Another distinctive advantage of employing Rh NPs is their role as promising contrast agents for X-ray fluorescence (XRF) bioimaging. We demonstrated the presence of Rh NPs within the whole 2PP-printed structure and emphasized the potential use of NP-loaded 3D-printed nanostructures for medical devices.

## Introduction

Additive manufacturing techniques gained significant attention in the last decades as an alternative to clean room fabrication techniques due to the possibility of layer-by-layer production of complex three-dimensional (3D) structures.^1−3^ Within this framework, two-photon polymerization (2PP) allows submicrometer resolution with relatively fast processing times.^4^

Photoresists play a crucial role in the 2PP process and determine the final intrinsic properties of the printed structures. Predominantly, photoresist formulations involve photoinitiators (PIs), monomers, oligomers, cross-linkers, and solvents as the diluent.^5,6^ Simultaneous absorption of two photons is achieved via a femtosecond laser with the center wavelength in the infrared spectrum. The initiation process starts with the PI within the photoresist absorbing the two-photon energy and converting it into radicals at the confined focal spot, called voxel.^7,8^ The formed radicals further activate the monomers in the propagation step for cross-linking, and the photopolymerization process is terminated when pairs of monomer radicals are combined.^8^ Finally, noncured regions are developed afterward with a proper solvent. The 2PP technique allows submicrometer resolution since the polymerization is only induced in the voxel when exceeding the specific threshold to form monomer cross-linking with two-photon excitation.^8^ For the above-described reasons, optimal 2PP photoresists should meet at least four requirements, including but not limited to having two-photon absorption close to the laser excitation wavelength, curing promptly at the voxel to prevent overheating, being optically transparent in the infrared range, and absorbing greatly in the UV region.^6,7^

Fabrication of high-resolution metal nanoparticle (NP)-embedded structures is drawing attention due to the possible range of applications from plasmonics and biosensors to flexible electronics.^9−13^ However, until now, the most prevailing strategy is in situ NP formation, i.e., the photoreduction of metal salts dispersed in the photoresist, so far characterized by limited control over the reduction yield of the metal salt as well as the formed NP size and morphology. Additionally, procuring homogeneous dispersions of metal salts is demanding due to low solubility limits in the photoresists. Thus far, a narrow amount of metal salt-mixed photoresists is examined for in situ photoreduction, i.e., gold and silver, often requiring higher laser powers than the bare photoresist.^12,13^ However, other noble metal salts such as Rh and Ru have not been investigated, as they might require even higher laser power to induce NP formation. The underlying reason can be ascribed to the colloidal NP formation mechanism in consideration of the standard redox potentials. For higher redox potential, the reduction requires less energy; thus, silver and gold ions, depending on the metal salt form, are more easily reduced than Rh and Ru salts.^14^ The alternative approach is embedding presynthesized metal NPs into the photocurable resin. However, metal NPs are known to interact with light by one- or two-photon absorption, and the absorbed energy is often dissipated in the form of heat, locally affecting the photoresist.^15^ In this approach, additional laser-induced impediments include the possibility of quenching converted radicals and scattering of the laser beam due to the presence of NPs.^15^ Herein, yet again, the 2PP printability of gold and silver NPs was highly explored, only achieving limited NP loading.^13,15,16^

Au and Ag NPs exhibit strong plasmonic properties in the visible and near-infrared regions;^17,18^ thus, the embedded metal NP concentration remains inevitably low, at around 0.01 and 0.006 v/v% for gold and silver NPs, respectively.^15^ In order to minimize complications arising from plasmonic behaviors, the NP size and morphology can be tuned. To our knowledge, despite the purposely tuned NP size, the integration of more than 0.01 wt % of gold NPs resulted in excess local heat generation due to photothermal effects.^13^ On the other hand, among the noble metals, Rh does not exhibit strong surface plasmon resonance (SPR) in the visible and near-infrared regions.^19^ In the interest of light-triggered interactions arising from 2PP due to the plasmonic responses in the visible and near-infrared range, Rh can be a conceivable alternative to increase the metal NP loading.

In this work, we addressed the challenge of loading high concentrations of metal NPs into resins for 2PP printing. We explored the effect of Rh NP integration on 2PP printing parameters, i.e., laser scanning speed and power. Under this scope, Rh NPs were synthesized, and their surface was functionalized to be dispersible within the photosensitive resist formulation. The surface-engineering process allowed the incorporation of up to 2 wt % Rh NPs. Therefore, we effectively showcased the integration of metal NPs at high concentrations. Additionally, capitalizing on being a contrast agent for X-ray fluorescence (XRF) imaging, we demonstrated the presence and uniform distribution of Rh NPs in the printed structures, highlighting the prospective use in diverse applications within the field of implantable medical devices.^20−22^

## Results and Discussion

### Nanoparticle Design

Rh NPs were synthesized by the polyol method^23^ and mixed with the photocurable resist, which consisted of *N*,*N*-dimethylacetamide (DMAc), 7-diethylamino-3-thenoylcoumarin (DETC), and pentaerythritol triacrylate (PETA) as the solvent, PI, and monomer, respectively. The reaction medium and the capping agent for the NP synthesis were deliberately chosen with the aim of increasing the dispersibility within the photoresist matrix. For this purpose, we exploited a well-established approach, i.e., the Hansen solubility model, in which absolute miscibility takes place only when the solubility values approach each other.^24,25^

As provided in Table 1, the Hansen solubility parameters of the individual photoresist components are 22.77 [(MPa)^0.5^] for the solvent and 16.20 [(MPa)^0.5^] for the monomer, as calculated in the previous studies. Following the data, the reaction solvent for Rh NP synthesis was selected as tetraethylene glycol (TEG) to have the closest parameter possible to PETA, and poly(vinylpyrrolidone) (PVP) was chosen as the capping agent. PVP’s Hansen solubility parameter varies between 17 and 33 [(MPa)^0.5^], depending on the referred literature, and the underlying reason behind this was ascribed to its average molecular weight, tacticity, and distribution, known to greatly affect the miscibility of polymers.^25^ Thus, in our studies, we chose high-average-molecular-weight (55 kDa) PVP to achieve effective miscibility both in the Rh NP synthesis reaction and in the final photoresist mixture.

**Table 1:** Hansen Solubility Parameter of the Photoresist and NP Synthesis Reaction Precursors/Solvents^a^

Substance	δ [(MPa)^0.5^]
PETA	16.20^26^
DMAc	22.77^27^
PVP	17.03^26^
	19.40^28^
	21.20^29^
	32.54^30^
EG	29.90^24^
DEG	24.80^24^
TREG	21.90^24^
TEG	20.30^24^

a EG: ethylene glycol. DEG: diethylene glycol. TREG: triethylene glycol.

**Figure 1:**
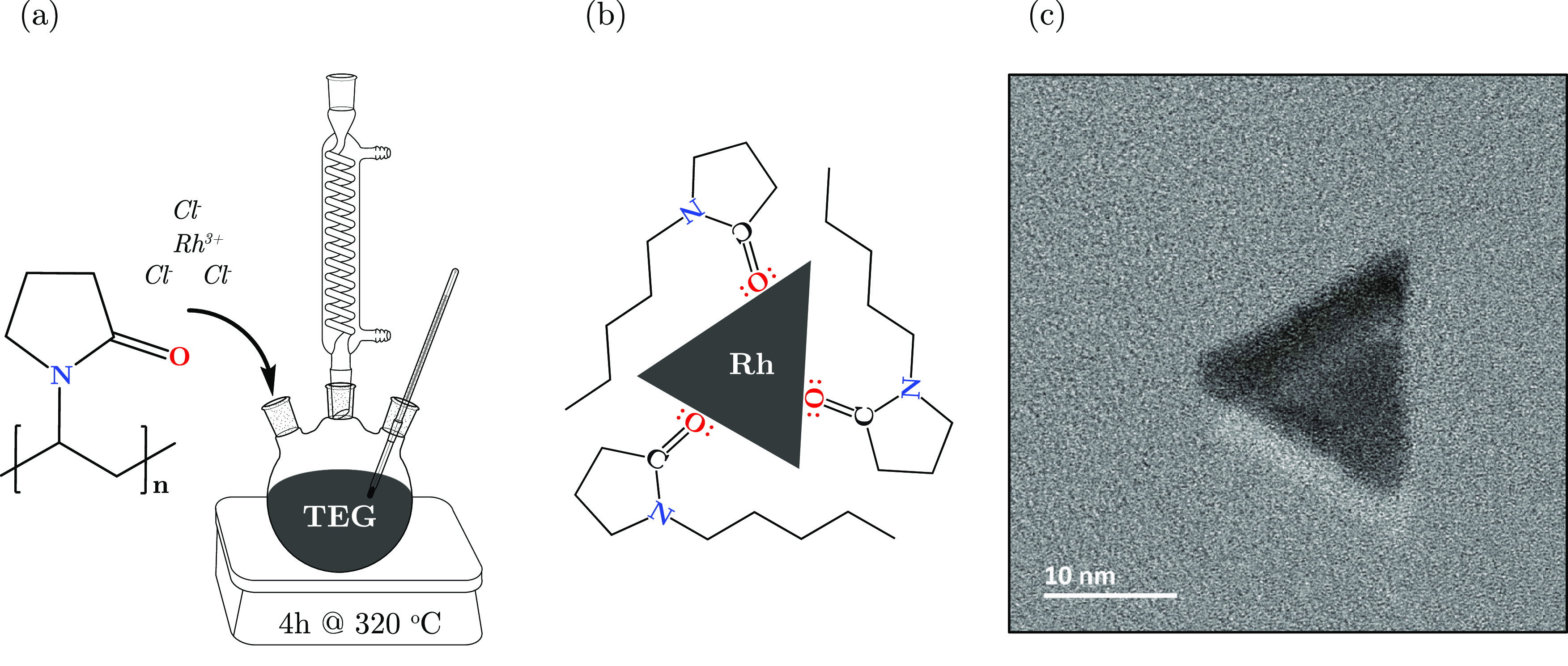
Schematic illustration of (a) the synthesis setup, (b) NP surface functionality, and (c) a representative TEM micrograph of Rh NPs.

**Figure 2:**
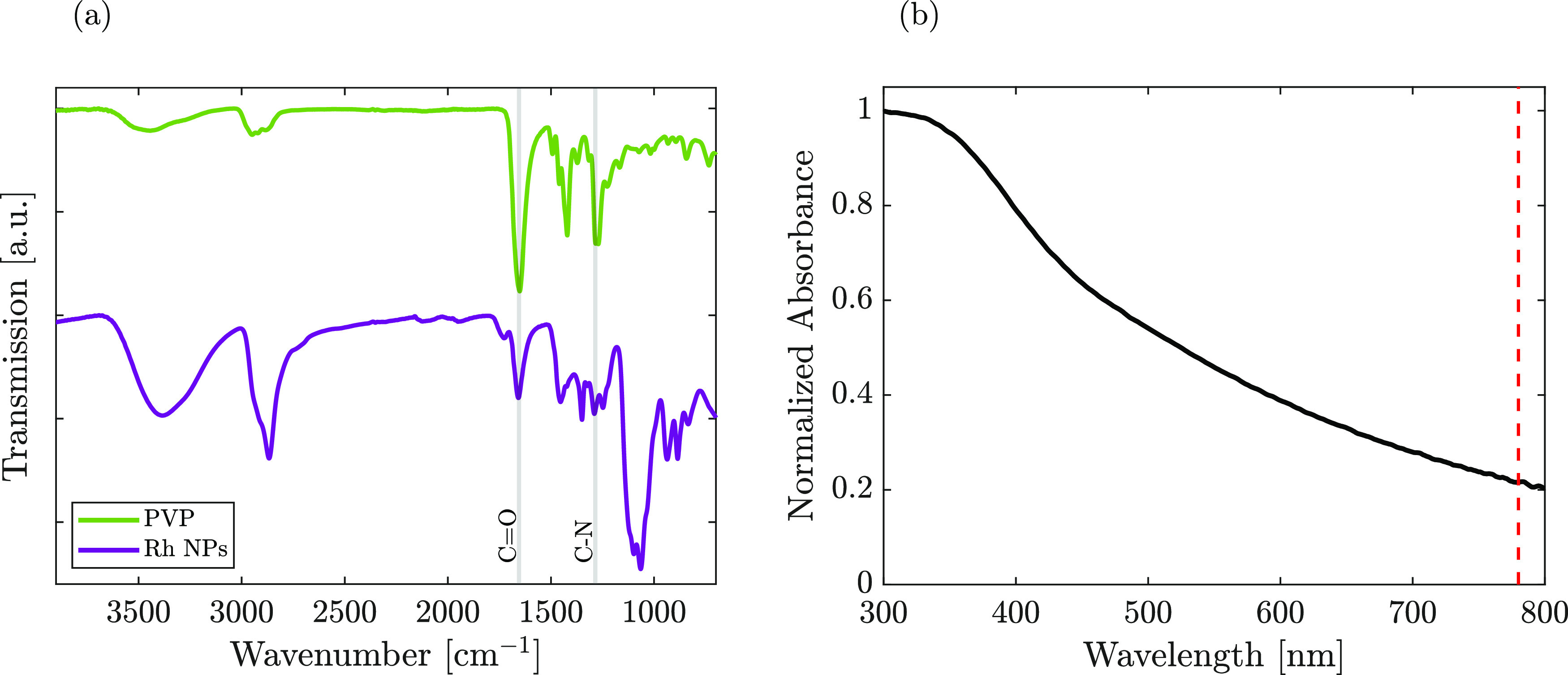
(a) FT-IR spectra of free PVP powder and PVP-capped Rh NPs. (b) UV–vis absorption spectrum of Rh NPs in DMAc; the red dashed line indicates the 2PP laser’s central wavelength.

The experimental setup and subsequent surface modification of the synthesized and surface-engineered Rh NPs are schematically provided in Figure 1a and 1b, respectively. The details regarding the surface functionalization were verified via Fourier transform infrared (FT-IR) spectroscopy analysis, presented in Figure 2a, revealing the successful PVP capping on the NP surface. Both the characteristic vibrational bands of C=O (1650 cm^–1^) and C–N (1283 cm^–1^) for the free PVP molecule were shifted when chemisorbed on the NP surface; in the case of the carbonyl group, the band shifted from 1650 to 1657 cm^–1^. The same observations are also valid for the C–N bond, which also slightly shifted from 1283 to 1289 cm^–1^. Shifts in the FT-IR spectrum indicated the coordination bond formation, occurring between the Rh surface atoms and oxygen/nitrogen groups of the PVP units.^31,32^ A magnified spectrum highlighting the two band shifts is reported in Figure S1. Furthermore, thermal gravimetric analysis (TGA) allowed quantitative measurements of the adsorbed and chemisorbed organic content and their relative weight compared to the inorganic core, as provided in Figure S2. The pyrolysis of the organic moieties was completed at 500 °C, and the Rh core content was estimated as 29 wt %.

The role of PVP was not only to function as a capping agent to prevent agglomeration but also to enhance the miscibility in the photoresist matrix. Nonetheless, PVP was proven to contribute to the morphology control of the metal NPs by binding specific planes, reducing the growth in that direction and enhancing along other directions, resulting in, i.e., triangular plates and spherical and decahedral shapes.^33,34^ Herein, the synthesized Rh NPs often exhibited a triangular morphology in the two-dimensional (2D) projection direction, as shown in Figures 1c and S2, with an average dry size (triangle height) of 17 ± 3 nm in transmission electron microscopy (TEM). In Figure S2, the selected-area electron diffraction (SAED) analysis evidenced the Rh face-centered cubic (fcc) crystal structure (ICDD PDF card 03-065-2866). The ring corresponding to the (022) plane exhibited the most intense integrated signal (Figure S2). It has previously been observed that the diffraction peak attributed to the (111) plane was the most intense with quasi-spherical Rh NPs.^31,35^ SAED has been used to investigate the role of PVP as the growth modulator in NP nucleation.^33,36^ Furthermore, the lowest full width at half-maximum (fwhm) of the peak associated with the (220) plane might indicate that the corresponding real-space crystallite size is predominant.^37^ Thus, we speculated that PVP capping with TEG as the solvent favored the growth of planes other than (111), such as the (022) plane.

In addition to dry size, colloidal size determination of the Rh NPs was crucial to understand the behavior in the suspended form, which was estimated as 106 ± 42 nm via dynamic light scattering (DLS) when dispersed in DMAc, as shown in Figure S3. When compared to the dry size, the obtained higher value for the colloidal size was ascribed to the additional contributions arising from the long-chain capping agent (PVP) and adsorbed solvent. The colloidal stability of the Rh NPs in DMAc was demonstrated by the evaluation of the mean count rate (MCR) variation between three consecutive DLS measurements, yielding a relative standard deviation of 1.54%, ascribed to random colloidal fluctuations rather than precipitation.^38^ Moreover, the obtained 0.32 ± 0.04 polydispersity index (PDI) further confirms the good dispersibility and narrow size distribution of the synthesized NPs in the given solvent, DMAc. The good dispersibility and colloidal stability were ascribed to the long-chain PVP (55 kDa) capping rather than NP morphology. Herein, we speculate that DMAc not only contributed to having better dispersion for Rh NPs but also facilitated the PI dissolution and prevented additional sonication/stirring/heating steps, which could be detrimental during the printing phase, due to the formation of local heat. The solvent was not evaporated after mixing since it does not contribute to polymerization,^12^ and its boiling point (165 °C) is high enough not to provoke its evaporation during the printing phase.

Prior to 2PP printing, the absorption spectrum, obtained by ultraviolet–visible (UV–vis) spectrophotometry of the synthesized Rh NPs, was evaluated in DMAc and is presented in Figure 2b. It exhibited a broad peak tailing in the entire visible region. Nevertheless, at the laser working wavelength (780 nm), we highlighted the absence of an SPR peak.

### Nanoparticle-Loaded Photoresist

To investigate the role of PIs and Rh NPs in affecting the printing parameters, different formulations were prepared, as summarized in Table 2. Furthermore, the same photoresist formulations were also tested without any NP addition as the control structures. It is important to note that the amounts of the monomer (PETA) and solvent (DMAc) were kept the same in all of the combinations to avoid secondary contributions.

**Table 2:** Photoresist Combinations

Sample ID	DETC [wt %]	Rh NPs [wt %]	PETA [mM]	DMAc [μL]
Bare-PI_0.125_	0.125	-	3.77	80
Bare-PI_0.5_	0.500	-	3.77	80
Bare-PI_2_	2.000	-	3.77	80
Rh–PI_0.125_	0.125	1.0	3.77	80
Rh–PI_0.5_	0.500	1.0	3.77	80
Rh–PI_2_	2.000	1.0	3.77	80

**Figure 3:**
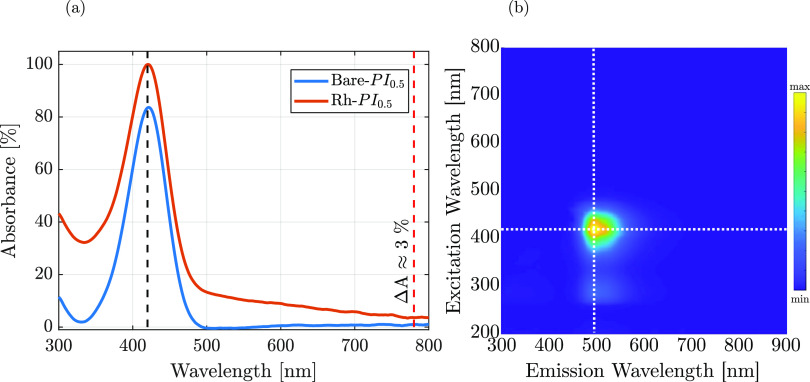
**(**a) UV–vis absorption spectra of Bare- and Rh–PI_0.5_ resists. The black dashed line highlights the PI absorption peak; the red dashed line evidence the relative absorbance variation (Δ*A*) between the Rh-incorporated and bare photoresist. The Bare-PI_0.5_ is used as the control. (b) 2D photoluminescence (PL) spectrum of the Rh–PI_0.5_ photoresist, highlighting the excitation and emission peaks with white dashed lines.

The PI, DETC, in the monomer and solvent mixtures, exhibited absorption and emission peaks at 420 and 490 nm, respectively, in all of the bare samples; a representative UV–vis spectrum from sample Bare-PI_0.5_ is provided in Figure 3a. The higher absolute intensity at 420 nm observed for Rh–PI_0.5_ was ascribed to the Rh NP absorbance contributions due to the presence of a broad peak tailing in the entire visible region (Figure 2b). The incorporation of Rh NPs did not alter the absorption peak position, and likewise, the fluorescent properties arising from the PI were preserved (Figure 3b). Even before printing, these results suggested the absence of detrimental plasmonic-related laser interactions, thus deviating from the existing literature mainly based on the use of Au NPs.^13,15^

### Printing Performance Evaluation

**Figure 4:**
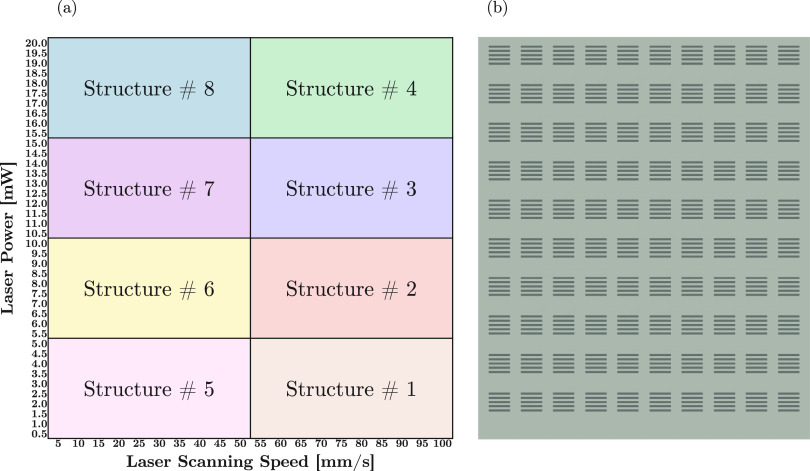
Representation of (a) matrix arrangement with a range of laser scanning speeds, 5–100 mm/s, and powers, 0.5–20 mW, consisting of (b) submatrix structures with a set of 5 lines for each printing parameter.

To evaluate the influence of the NP addition on the printing parameters, several laser scanning speeds and powers were investigated through a matrix structure. The matrix was constituted of 8 separated submatrix structures to avert extreme laser gradients in a single matrix. The submatrix structures were designed to have dimensions less than the field of view limit (230 μm), in accordance with the manufacturer’s instructions for a 60× objective lens in oil (VAT mode), to prevent stitching artifacts. A schematic representation of the full matrix is given in Figure 4a. Each submatrix structure (Figure 4b) was composed of combinations of 10 different laser scanning speeds and powers. For instance, in submatrix structure #2, laser power and scanning speeds varied from 5.5 to 10.0 mW and 55 to 100 mm/s, respectively. The printing quality of each power and scanning speed was evaluated with 5 individual lines printed with dimensions of 10 μm × 1 μm × 2 μm (*L* × *W* × *H*). Visual details regarding the dimensions of the printed lines are provided in Figure S3.

**Figure 5:**
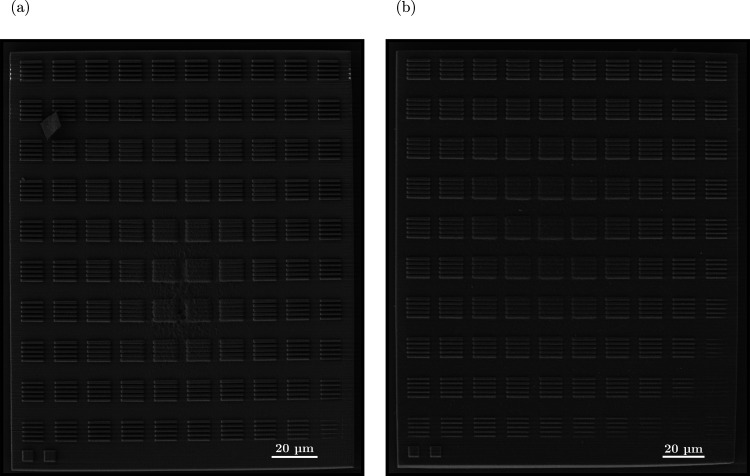
SEM micrographs of 2PP-printed submatrix #2 for (a) Bare-PI_0.5_ and (b) Rh–PI_0.5_ formulations.

The observations made with scanning electron microscopy (SEM) on submatrix structure #2 (Rh–PI_0.5_ vs Bare-PI_0.5_) revealed that one of the laser-induced effects, namely, scattering, was negligible even when introducing Rh NPs (Figure 5). This claim was further supported by the absence of visible scattering artifacts in the video recorded during the printing session of the base for the submatrix structure using the Rh–PI_0.5_ formulation (Movie M1). Additional SEM images constituting the whole matrix structure for Rh– and Bare-PI_0.5_ resists are provided in Figures S4 and S5, respectively.

To further investigate the printing performance of each resist combination, SEM micrographs were employed for the qualitative evaluation of the printing quality when polymerizing line sets through the observation of their damage and polymerization parameters.

The printing dynamic range (DR) of the structures was estimated with the following formula and implemented in Table 3, where *P*_polymerization_ and *P*_damage_ indicate the power required to polymerize and damage, respectively.^39^
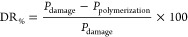


**Table 3:** Dynamic Range (DR) of the Resist Formulations, Including the Polymerization and Damaging Powers at 50 and 100 mm/s Scanning Speeds

Sample	*P*_polymerization_ [mW]	*P*_damage_ [mW]	Scanning speed [mm/s]	DR [%]
Bare-PI_0.125_	10.5	>20	100	-
	7.5	15.5	50	51.6
Bare-PI_0.5_	6.5	11.5	100	43.5
	4.5	12.0	50	62.5
Bare-PI_2_	5.5	11	100	50.0
	5.0	11	50	54.5
Rh–PI_0.125_	13.0	>20	100	-
	10.5	>20	50	-
Rh–PI_0.5_	7.5	15.5	100	51.6
	7.0	17.0	50	58.8
Rh–PI_2_	7.5	15.5	100	54.5
	4.0	10.5	50	61.9

The damage power was identified as the minimum power leading to detrimental effects that occurred on the printed structures, such as not well-defined line sets, interline connections, and locally burnt materials. Examples of adverse effects arising from extreme printing conditions could be observed in the micrographs presented in Figures S4 and S5, particularly in panels (g) and (h), wherein printing artifacts became evident. On the contrary, the power required to polymerize was selected when enough polymerization was achieved. Under this scope, the printing performance at two different scanning speeds, 50 and 100 mm/s, were examined. It is crucial to highlight that the feature thickness was not considered in the DR estimation. Regarding the Bare- and Rh–PI_0.125_ samples, damaging laser powers and the DRs were not determined since the matrix structures were designed and set up to a maximum of 20 mW power to ensure a high cost efficiency for a possible large-scale translation. Furthermore, maintaining minimal laser powers averted redundant variables for achieving optimal printing.

As expected, a higher concentration of the PI led the polymerization to start with lower powers, in both the NP-loaded and bare formulations (cf. Table 3). Printing in the presence of Rh NPs shifted the power values for both polymerization and damage while not adversely impacting the DR values. The underlying reason behind this could be identified by the analysis of Rh NPs with PL and UV–vis data. No significant peak was observed at 780 nm, as shown in Figure 2b. However, there was a non-negligible absorption that might have contributed to an inefficient initiation of photopolymerization in the resist formulations (Figure 3a), justifying the need for higher power and/or lower speed compared to the bare resist formulations. In fact, although Rh NPs do not possess plasmonic peaks in the visible and near-infrared ranges, the residual absorption could be identified as responsible for the consumption of extra laser power. This outcome was expected since the laser light interacts in the form of scattering or absorption.^15^ The negligible laser scattering and absence of SPR peak identified one-photon absorption as the most likely interference mechanism, which was conceivably dissipated in the form of heat (nonradiative transitions). The amount of NP absorption affects the printing quality; for instance, high local heat generation might lead to bubble formation. The relative absorbance percentage in the presence of 1 wt % Rh NPs was calculated as ≈3% at the laser working wavelength (Figure 3a). This low value demonstrated the possibility of having a high Rh NP concentration in 2PP photoresists compared to SPR-exhibiting NPs. The resulting heat dissipation could be influenced by other factors including but not limited to the employed printing speed. Regarding the shift in polymerization initiation in the presence of NPs, there were two other underlying causes besides absorption. The presence of NPs introduced extra surface areas into the system, because of their high surface-to-volume ratio. That might have created trap sites for the PI preventing them from reaching monomer species to start the polymerization. Second, the embedded NPs might have also quenched the formed radical species via PIs, when interacting with their surface.^15^

The analysis of the printed structures was divided into two steps. The first step consisted of selecting the PI concentration among the three tested concentrations. The second step focused on the evaluation of the printed line set accuracy compared to the computer-aided design model, aiming at identifying the optimal scanning speed and laser power.

From the data presented in Table 3 and morphological evaluations on SEM micrographs, the best photoresist formulations were identified as the Bare- and Rh–PI_0.5_, and their optimal printing conditions were found in submatrix structure #2. The lowest PI concentration (0.125 wt %) was excluded because it required higher laser powers and, thus, higher costs to initiate polymerization (Table 3). The highest tested PI concentration (2 wt %) resulted in not well-defined line sets. SEM images of Bare-PI_0.125_ and Bare-PI_2_ resists and Rh NP-embedded counterparts of them are presented in Figure S6 at their optimal printing conditions. It is worth noting that the chosen DMAc volume did not impede successful layer-by-layer printing with any of the prepared formulations (Movie M1). Moreover, resin drag effects^4,39^ were not observed during the printing process upon stage motion.

**Figure 6:**
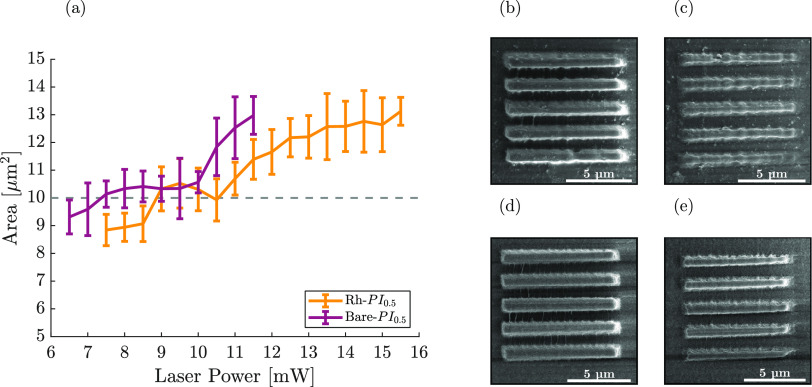
(a) Area estimation of the printed structures. The reported values are the averages from quintuplicates. The dashed line represents the actual designed area (10 μm^2^). SEM micrographs of the printed lines at (b) 9.5 mW with Rh–PI_0.5_ and (d) 8 mW with Bare-PI_0.5_. SEM micrographs of line sets just under the polymerization threshold were obtained at (c) 7 mW with Rh–PI_0.5_ and (e) 6 mW with Bare-PI_0.5_.

Further analysis of the optimal printing parameter was conducted for the chosen photoresist formulations via the structural analysis of the printed line sets through SEM micrographs in Figures 5, S4, and S5. When the scanning speed was fixed to 100 mm/s, the line area with respect to the actual design was analyzed. The designed area of the lines was fixed to 10 μm^2^, and the results from the estimation of the printed structures are shown in Figure 6a, where bare formulation gave the desired area between 7.5 and 10 mW laser power. Besides, formulation with Rh NP incorporation yielded the optimal printing power at the 8.5–11 mW power range. Among these values, the ideal printing powers were found as 8 and 9.5 mW for Bare-PI_0.5_ and Rh–PI_0.5_ resists, respectively, magnified in Figure 6d,b. For examples of structures printed under the polymerization threshold, SEM micrographs were presented for structures printed at 7 and 6 mW for Rh– and Bare-PI_0.5_ formulations, respectively (Figure 6c,e).

Furthermore, the interaction between Rh NPs and the photosensitive resist was probed with FT-IR analysis, and the spectra are presented in Figure S7. The PI was also curable with UV light, which allowed for the polymerization of a cm-sized film for FT-IR analysis. The chemical bond formation, in terms of polymerization in acrylic resin formulations, would be the same. Thus, the ink formulations were cured using a UV lamp instead of 2PP, as the FT-IR technique requires large samples for the analysis. Rh– and Bare-PI_0.5_ resists were analyzed after curing as a solid film. Characteristic vibrational bands of acrylic resins C=O and C=C were identified in both samples, located at 1720 and 1635 cm^–1^, respectively.^40^ In order to have a solid understanding of the interaction mechanism of Rh NPs, the FT-IR spectra were normalized to the C=O band from PETA since it is known to be unaltered upon polymerization.^41^ On the other hand, C=C bonds open during polymerization and convert into C–C.^41^ When analyzing the two UV-polymerized films (Bare- and Rh–PI_0.5_), the absence of significant shifts in the vibrational bands (C=C and C–C) implied the successful integration of Rh NPs in polymerized PETA chains. The presence of Rh NPs was evidenced by the C=O stretching vibration of PVP capping on the NPs. Compared to Rh NPs (1657 cm^–1^), the band shifted to a lower value (1651 cm^–1^) in Rh–PI_0.5_ due to interactions occurring between NPs and photoresists after UV curing.

**Figure 7:**
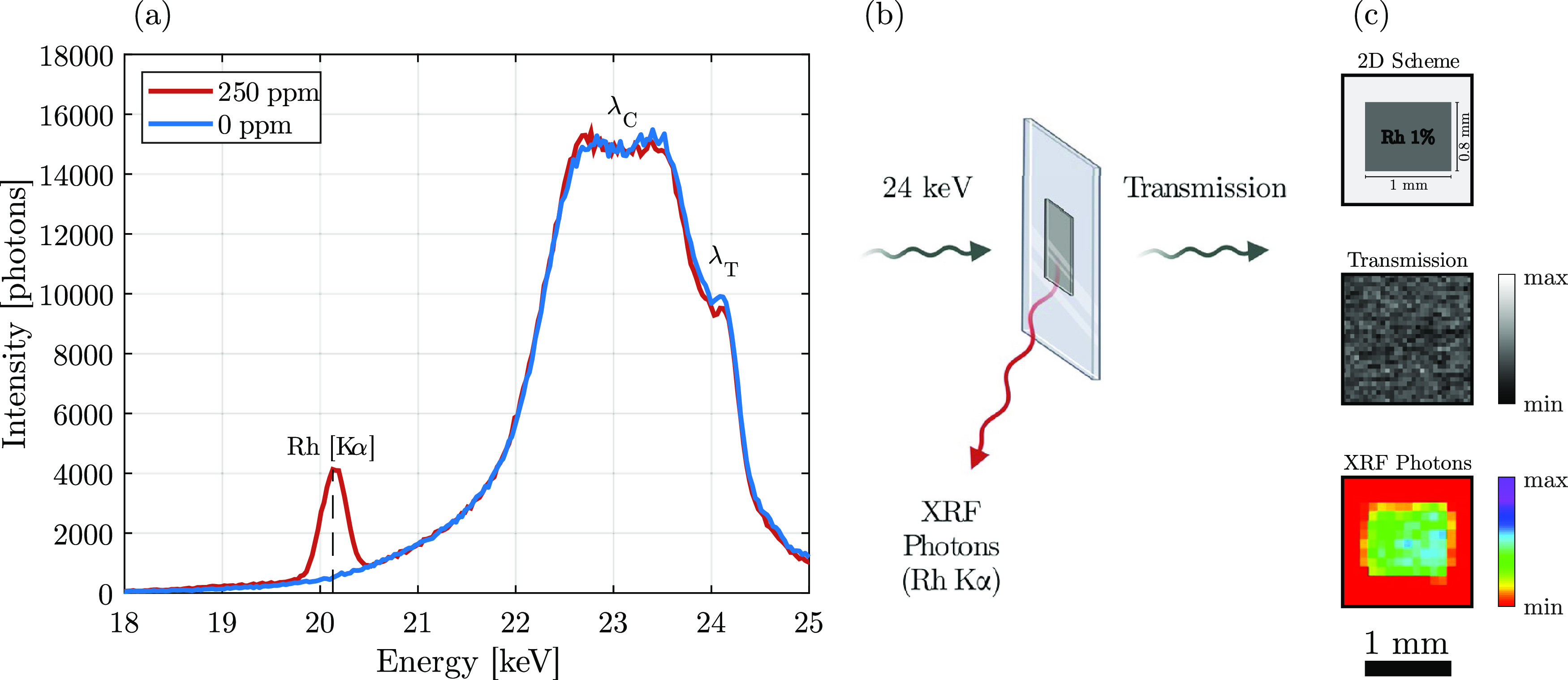
(a) X-ray fluorescence (XRF) spectrum recorded for a vial containing Rh NPs dispersed in a solution (red) compared to the control (blue). (b) Schematic representation of the XRF imaging setup. (c) 2D scheme and projection images (transmission vs XRF) of a thin 2PP-printed film using the Rh–PI_0.5_ resin.

In recent years, XRF was demonstrated to be employable for molecular imaging in vivo, using metal liquid jet as the X-ray source (24 keV) and contrast agents containing elements matching their absorption edge (core electron excitation) with the source energy, e.g., Rh, among others.^20,22,31^ In Figure 7a, the X-ray spectrum collected by the XRF detectors when scanning a vial was presented. In the vial containing the resin with dispersed Rh NPs, it was possible to detect the Rh Kα radiation (20.2 keV) originating from Rh NPs, contrarily to control. Thomson scattering (λ_T_) and Compton scattering (λ_C_) are also indicated. The successful integration of Rh NPs in the resin could enable XRF imaging of the 2PP-printed structures. A thin film was 2PP-printed using the Rh–PI_0.5_ resist (cf. Table 3) on a glass substrate and vertically positioned in the XRF imaging setup (Figure 7b). The transmitted X-ray and XRF photons were used to generate two projection images (Figure 7c). While the sensitivity in transmission was not sufficient to detect the 2PP-printed layer due to the 20 μm thickness, the XRF signal demonstrated and highlighted the presence of Rh NPs within the whole printed area (1 mm × 0.8 mm). Considering the colloidal stability of the NP dispersion evidenced through the MCR variation studies, we speculate that Rh NPs were uniformly distributed. Further analyses employing, e.g., small-angle X-ray scattering (SAXS) and, in particular, grazing incidence SAXS could confirm these claims.^42,43^ Furthermore, SAXS has recently been used to demonstrate that the photoinitiated polymerization process might trigger chemically induced nanostructural changes.^44^ Due to the high elemental specificity of XRF imaging,^31^ its use with 2PP-printed Rh-containing microstructures within the biomedical field could pave the way to the design of novel, functional implantable medical devices, potentially allowing the study of structural shape changes and NP release over time in vivo.

Finally, it is important to highlight that the limited laser scattering resulting from plasmonic-related interactions permitted the incorporation of Rh NPs in much higher concentrations than other formulations in the existing literature for 2PP printing.^12,13,15^ Likewise, surface-engineering NPs according to the photoresist formulations also allowed the loading of Rh NPs at much higher concentrations. After the successful incorporation of 1 wt % NPs, the concentration was further increased to 2 wt %. An SEM micrograph of the 2PP-printed structure with Rh–PI_2_ (2 wt % Rh) is provided in Figure S7. Lower PI concentrations were also tested but were not sufficient to initiate the polymerization process (data not shown). Although the printing was successful with Rh–PI_2_ in terms of light-triggered interactions and dispersibility in the photoresist formulation, further development in the 2PP printing system would be required. The addition of a higher concentration of NPs affected the light transmission in the visible range, i.e., the vision through the resin, which is highly dependent on the photoresist thickness and shows exponential behavior following the Beer–Lambert law.^45^ This phenomenon made identification of the substrate–resin interface troublesome for correct polymerization initiation. The integration of an extra auxiliary lamp in the 2PP setup could increase the brightness and might solve the visibility problem, eventually allowing a further increase in the NP concentration.

The obtained loading of ex situ-formed metal NPs into the resin for 2PP printing (up to 2 wt %) was achieved by circumventing the limitations imposed by plasmonic interferences observed when loading Au and Ag NPs,^15^ leading to thermal damage on the printed structures already when using Au concentrations higher than 0.01 wt %.^13^

## Conclusions

In this work, we presented a strategy to integrate Rh NPs with high concentrations of up to 2 wt % in the 2PP-printed structures by manipulating their surface using the principles of Hansen solubility parameter to enhance the compatibility with the photocurable resin formulations. The element selection (Rh) was deliberately made to minimize the interactions between plasmon-resonance absorption and the 2PP laser. This proved to be successful with a relative absorption of only 3%. Moreover, the NP integration in the photocurable resin did not alter the fluorescence and absorption peak positions of the PI. Among different resist formulations, a PI concentration of 0.5 wt % yielded a structure containing the optimal printing parameter for both Rh NP-loaded and bare formulations. Quantitative printability studies revealed minimal variations with 1 wt % Rh NPs compared to the bare control. We observed a shift in the polymerization threshold upon introduction of Rh NPs. The optimal printing power at 100 mm/s was determined as 8 and 9.5 mW for the Bare- and Rh–PI_0.5_ resist, respectively. Furthermore, we also demonstrated the possibility of printing structures with 2 wt % NP loading but concluded the need for further improvement in the 2PP system. Finally, by exploiting XRF imaging, we verified the presence of Rh NPs within the 2PP-printed structures and suggested potential implementation in medical devices. These studies pave the way for the design and fabrication of functional devices with complex 3D microstructures.

## Experimental Section

### Materials

Rhodium(III) chloride hydrate (RhCl_3_·*x*H_2_O, Rh 38.5–45.5%), Tetraethylene glycol (TEG, HO(CH_2_CH_2_O)_3_CH_2_CH_2_OH, >99%), poly(vinylpyrrolidone) (PVP, C_2_H_2_N(C_6_H_9_NO)_*n*_C_13_H_10_NS_2_, average MW = 55 kDa), 7-(diethylamino)-3-(thiophene-3-carbonyl)-2*H*-chromen-2-one (DETC), and 3-(trimethoxysilyl)propyl methacrylate (TMPSM, H_2_C=C(CH_3_)CO_2_(CH_2_)_3_Si(OCH_3_)_3_, 98%) were obtained from Sigma-Aldrich, Sweden. Pentaerythritol triacrylate (PETA, C_14_H_18_O_7_, stab. with 300–400 ppm 4-methoxyphenol) was obtained from Thermo Fisher Scientific, Sweden. Solvents, including ethanol, acetone, *N*,*N*-dimethylacetamide (DMAc, CH_3_CON(CH_3_)_2_), and 2-propanol were of analytical grade and were obtained from Sigma-Aldrich, Sweden. Acetic acid (CH_3_CO_2_H, ≥99.85%) was glacial and was also purchased from Sigma-Aldrich, Sweden. All chemicals were used without further purification.

### Nanoparticle Synthesis

Rh NPs were synthesized via a polyol method using TEG as the main solvent. Briefly, RhCl_3_ (0.5 mmol) and PVP (10 mmol, repeating units) were dissolved in a mixture of DI water (0.5 mL) and TEG (20 mL) in a three-neck flask while magnetically stirring. The solution was heated up to 120 °C and kept for 15 min to evaporate water. Subsequently, the system was brought to the refluxing temperature (320 °C) and let react for 4 h. The dispersion color turned from red-brown to black. Subsequently, the system was cooled down to room temperature. The dispersion was then collected, and the as-synthesized Rh NPs were stored at 4 °C, until further use.

### Photoresist Preparation

1 wt % as-synthesized Rh NPs, with respect to the PETA monomer, were washed with acetone in a 1:9 (Rh NP stock/acetone) ratio. Centrifugation is conducted for 5 min at 6000 rpm and 15 °C. The washed, as-synthesized Rh NPs were then collected with 50 μL of DMAc solvent to favor the NP dispersion, which was further promoted by a few minutes of sonication and vortexing. The PETA monomer (3.77 mM) was separately mixed with DMAc (10 μL) and combined with an NP+DMAc suspension. Corresponding initiator amounts, 0.125, 0.5, and 2.0 wt %, were dispersed in 20 μL of DMAc and added to the monomer–NP mixture prior to the printing process. The prepared photoresists were stored at room temperature in a dark environment.

For the bare photoresist formulations, which were adapted from a previous literature, 3.77 mM of PETA monomer was mixed with 60 μL of DMAc solvent.^12,13,46^ An extra 20 μL of DMAc was used to disperse the photoinitiator just before the printing session. The total DMAc volume (80 μL) was equal to the volume used for the NP-containing formulations. The prepared samples were also stored in a dark environment at room temperature.

### 2-Photon Printing Process

Prior to the 2PP printing, glass substrates were surface-treated with oxygen plasma (Diener electronic GmbH + Co. KG, Ebhausen, Germany) and silanized to achieve improved adhesion of the printed structures. Briefly, silanization was performed in a 1:1 ethanol/DI water volume ratio mixture while mechanically stirring. Subsequently, 300 μL of acetic acid was pipetted and 2 mL of silane coupling agent TMPSM was added dropwise to the stirring solution and allowed to react for 20 min. Afterward, oxygen plasma-treated glass substrates were covered with the silane coating solution and kept for 30 min. Following the treatment, each glass substrate was rinsed with 2-propanol and DI water.

All of the submatrix structures were 2PP-printed using the high-resolution 3D printing system, NanoOne (UpNano GmbH, Vienna, Austria), with custom-made photoresist combinations either with Rh NPs or bare formulations. Submatrix structures were modeled, and the printing parameters, such as alternating laser powers and scanning speeds, were implemented via THINK3D software (UpNano GmbH, Vienna, Austria). Structures were printed on 10 mm × 10 mm glass substrates employing VAT mode using a 60× objective lens in oil immersion with a 1.42 numerical aperture and 0.15 mm working distance. For the print settings, fine infill and conservative slicing modalities were applied. To avoid potential printing errors in the line structures arising from the inexact glass–monomer interface determination, submatrices were designed and printed on a 10 μm thick base: printing power and laser scanning speeds were 10 mW and 100 mm/s, respectively, for all of the resist formulations except for the low PI-containing formulations printed with 20 mW power. Single submatrix structures took less than 2 min of print time.

After the printing, nonpolymerized portions of the structures were developed by three consecutive 2-propanol baths, with 10 min of rinsing.

Printing for XRF imaging was conducted the same as for the matrix structures on a microscope glass slide with dimensions of 40 mm × 26 mm × 1.1 mm (*L* × *W* × *H*) to minimize the absorption of the X-rays via the glass.

### Characterization Techniques

Rh NPs were morphologically and structurally characterized by several techniques. Transmission electron microscopy (TEM, JEM-2100F, 200 kV, JEOL) was employed to evaluate the morphology and dry size of the Rh NPs. Values for the average and standard deviation were obtained by counting at least 200 Rh NPs from several micrographs with different field of views. Drop-cast copper grids were used for TEM. Scanning electron microscopy (SEM, ZEISS LEO 1550) was used to analyze the printed matrix structures. After the development step, samples were air-dried and mounted on an SEM holder via carbon conductive tabs. The printed structures were coated with a Au/Pd layer (a few nanometers) using a sputter coater (Polaron SC7640) to avoid charge artifacts. Several acceleration voltages were chosen for SEM imaging, ranging from 7 to 10 keV. Dynamic light scattering (DLS) was performed to estimate the hydrodynamic size, polydispersity index (PDI), and mean count rate (MCR) to evaluate the Rh NP properties in DMAc, using the Zetasizer Nano ZS90 system (Malvern, U.K.). Given the low PDI values, number-average values and related standard deviations were used and compared to the dry (TEM) size. The Rh NP crystallographic phase was determined using selected-area electron diffraction (SAED), with TEM. The presence of the capping agents and the absorption mechanisms on the NP surface were investigated with a Fourier transform infrared spectrometer (FT-IR spectrometer, Thermo Fisher Scientific), in ATR mode. FT-IR spectra of the UV-cured films were also acquired. An ultraviolet–visible spectrophotometer (UV–vis spectrophotometer, NP80, Implen) and PL (spectrofluorometer, FP-8300, Jasco) were used to evaluate the photophysical properties of Rh NPs, PI, and their mixture. The quantification of the organic content on the Rh NP surface was achieved with a TGA instrument (TGA550, TA Instruments). The Rh concentration was estimated with XRF analysis (Rh Kα), with diluted dispersions of Rh NPs, Rh standard solution (1000 ppm), and water in 2 mL centrifuge tubes. The acquired X-ray spectra were then utilized to estimate the NP concentration normalized to the Rh standard.^21^

### X-ray Fluorescence Imaging

A projection image of a thin 2PP-printed structure, fabricated using a Rh–PI_0.5_ resist on a glass substrate and vertically positioned in XRF, was made. The step size was set to 50 μm and the exposure time was set to 1 s in both *x* and *y* directions. Binning (2 × 2) by average was performed to reduce the noise. The XRF projection (color) was compared to the X-ray absorption projection image (grayscale). Further details about the X-ray source, detectors, and setup were described in our previous works.^47^

## Supporting Information Available

Detailed results from the characterization of synthesized NPs, designed submatrix structures of the photoresist formulations, and additional analysis on UV-cured films (PDF)
2PP printing movie: base printing of the submatrix structure using Rh–PI_0.5_ resist formulation (MP4)

## Abbreviations

2PP: two-photon polymerization
NP: nanoparticle
SPR: surface plasmon resonance
XRF: X-ray fluorescence
PI: photoinitiator
DMAc: *N*,*N*-dimethylacetamide
DETC: 7-diethylamino-3-thenoylcoumarin
PETA: pentaerythritol triacrylate
TEG: tetraethylene glycol
PVP: poly(vinylpyrrolidone)
FT-IR: Fourier transform infrared
TGA: thermal gravimetric analysis
TEM: transmission electron microscopy
SAED: selected-area electron diffraction
fcc: face-centered cubic
fwhm: full width at half-maximum
DLS: dynamic light scattering
PDI: polydispersity index
MCR: mean count rate
UV–vis: ultraviolet–visible
PL: photoluminescence
SEM: scanning electron microscopy
DR: dynamic range
TMPSM: 3-(trimethoxysilyl)propyl methacrylate
