# Supplementary material for: Two-Photon Polymerization Printing with High Metal Nanoparticle Loading
Source: ACS Appl Mater Interfaces. 2023 Oct 10;15(42):49794–804. doi: 10.1021/acsami.3c10581 (PMC10614202; doi:10.1021/acsami.3c10581)
Supplement: Supplementary file 1 — am3c10581_si_001.pdf [file am3c10581_si_001.pdf]

Supporting Information

Two-photon Polymerization Printing with High Metal Nanoparticle Loading

Nuzhet I. Kilic[ab], Giovanni M. Saladino[b]*, Sofia Johansson[c], Rickard Shen[d], Cacie McDorman[e], Muhammet S. Toprak[b], Stefan Johansson[a]*

[a]Department of Materials Science and Engineering, Microsystems Technology, Uppsala University, SE 75103 Uppsala, Sweden

[b]Department of Applied Physics, Biomedical and X-Ray Physics, KTH Royal Institute of Technology, SE 10691 Stockholm, Sweden

[c]Department of Materials Science and Engineering, Biomedical Engineering, Science for Life Laboratory, Uppsala University, SE 75103 Uppsala, Sweden

[d]Kanthal AB, SE 73427 Hallstahammar, Sweden

[e]Alleima Advanced Materials, FL 32164 Palm Coast, United States

*Corresponding Authors: inci.kilic@angstrom.uu.se; saladino@kth.se

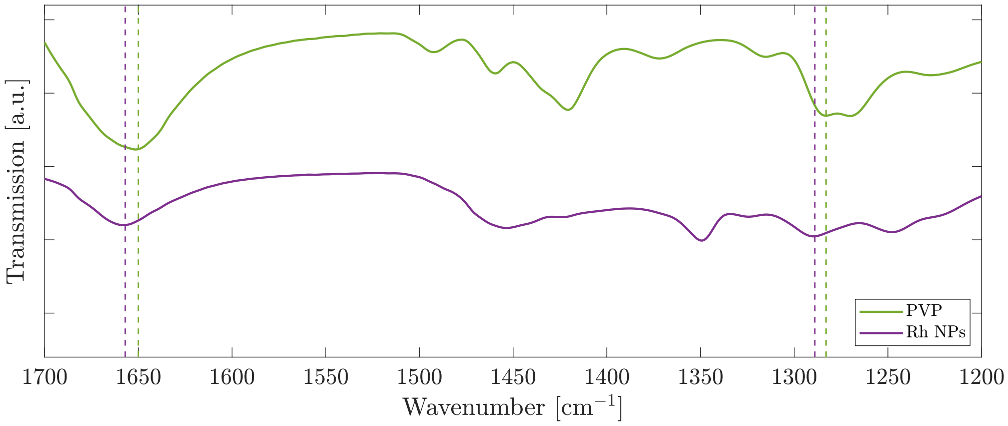

Figure S1. Magnified FT-IR spectra of free PVP powder and PVP-capped Rh NPs, highlighting the C=O and C-N vibrational band shifts.

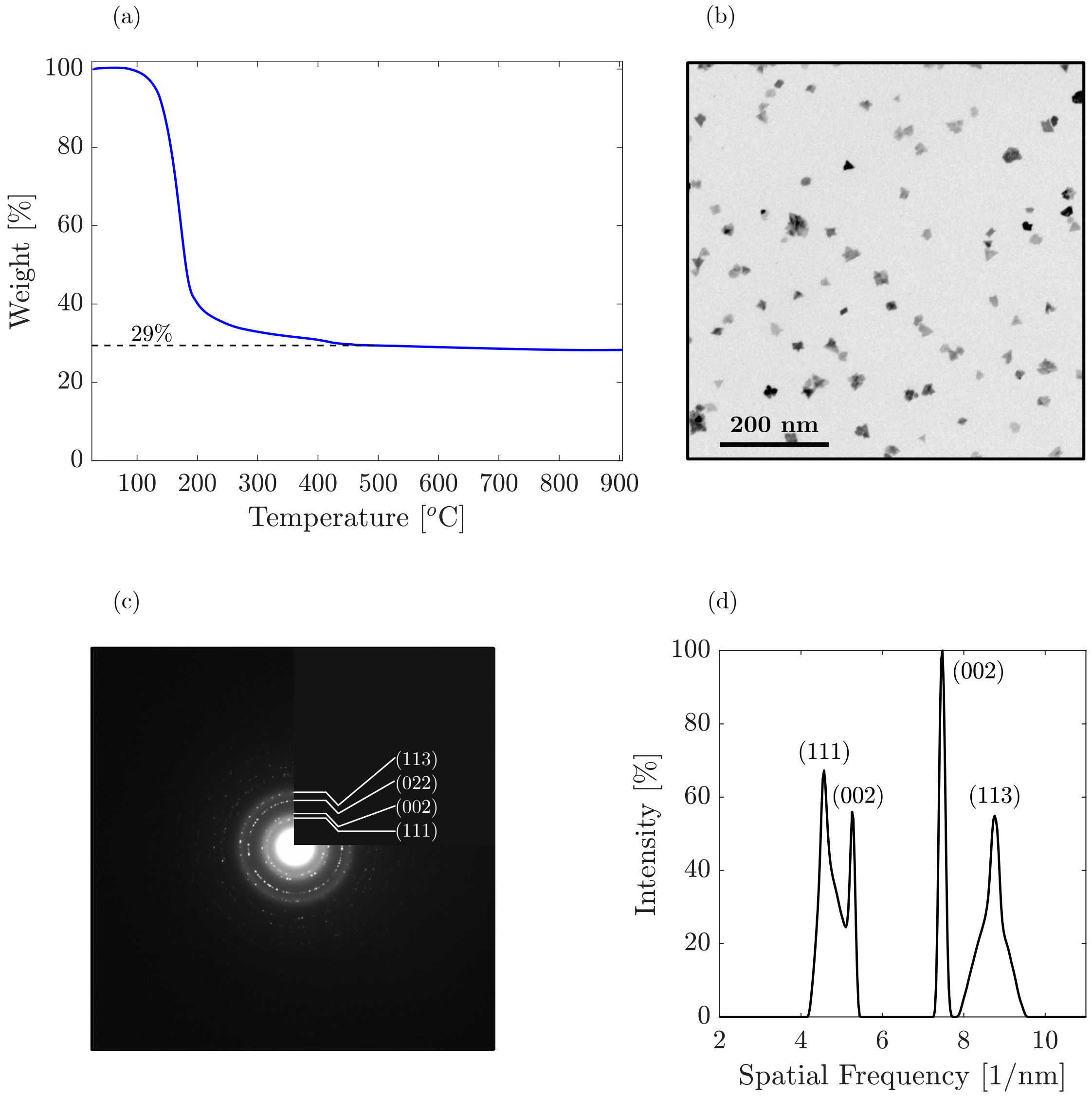

Figure S2. (a) TGA for inorganic and organic moieties determination in the synthesized Rh NPs; the dashed line at 500 ºC represents the Rh inorganic content (29 wt.%). (b) TEM micrograph of Rh NPs. (c) SAED analysis for crystal structure determination with the highest crystal plane intensities. (d) SAED integrated profile, highlighting the planes corresponding to the diffraction peaks.

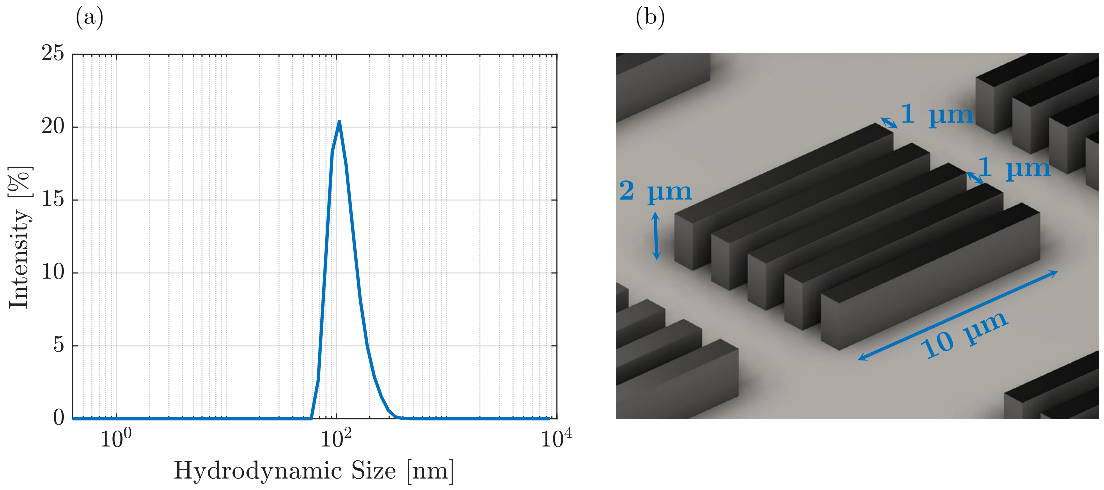

Figure S3. (a) Colloidal size distribution of Rh NPs in DMAc via DLS. (b) Dimensions of the printed line sets with 10 µm x 1 µm x 2 µm (L x W x H) and 1 µm spacing.

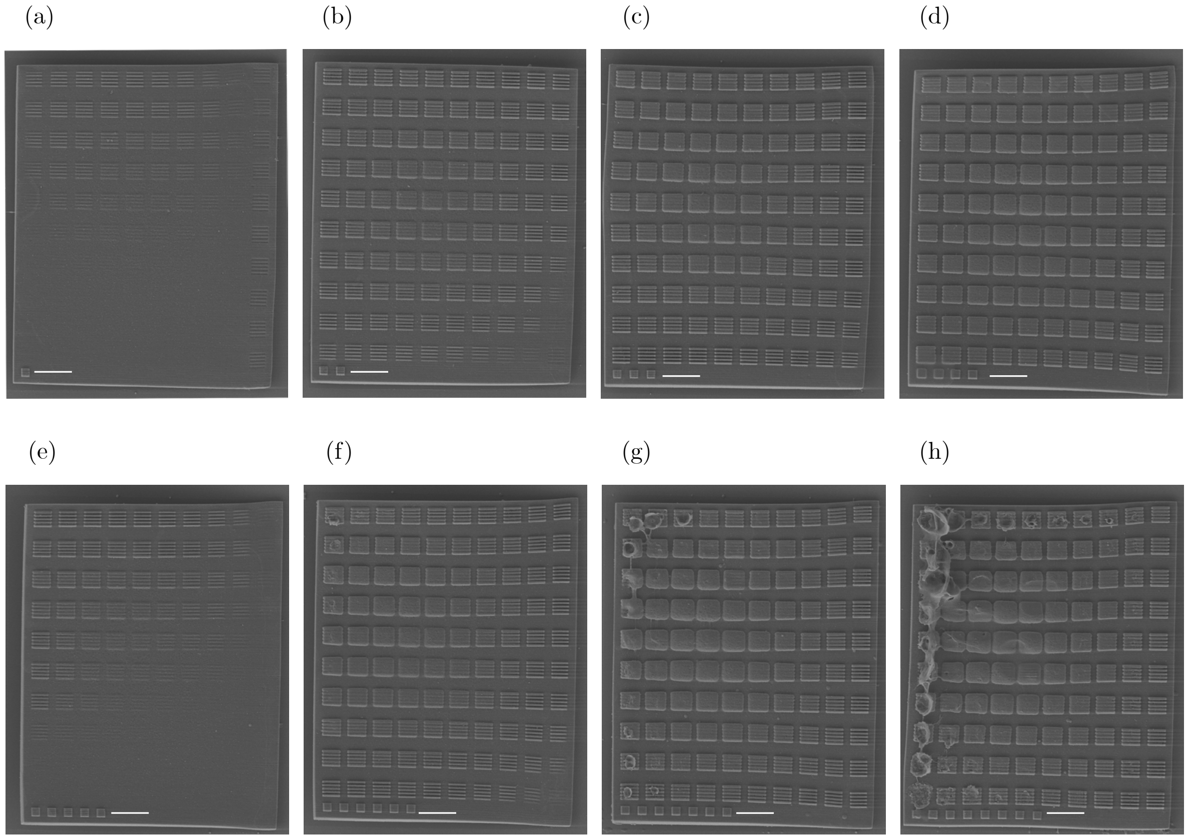

Figure S4. SEM micrographs of 2PP-printed Rh-$PI_{0.5}$ samples; from (a) to (h) showing the submatrix structures from #1 to #8, respectively. All the scale bars are 20 μm.

(a) (b) (c) (d) (e) (f) (g) (h)

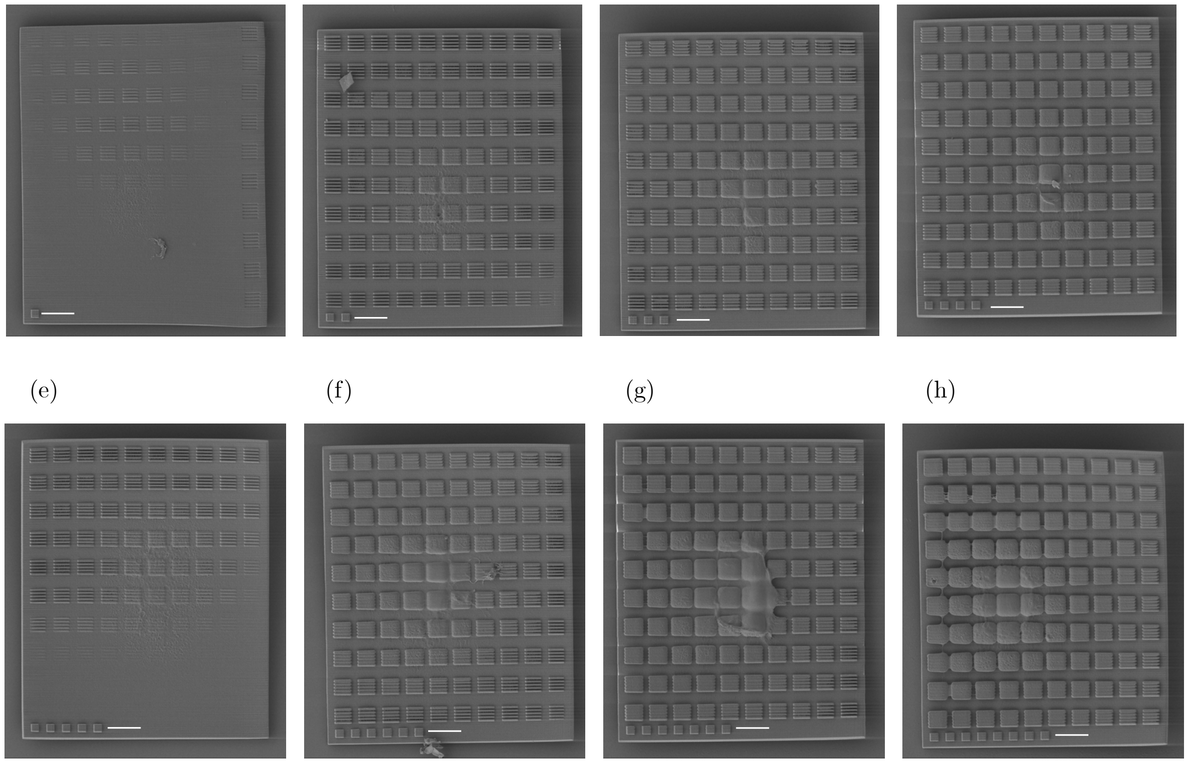

Figure S5. SEM micrographs of 2PP-printed Bare-PI$_{0.5}$ samples; from (a) to (h) showing the submatrix structures from #1 to #8, respectively. All the scale bars are 20 μm.

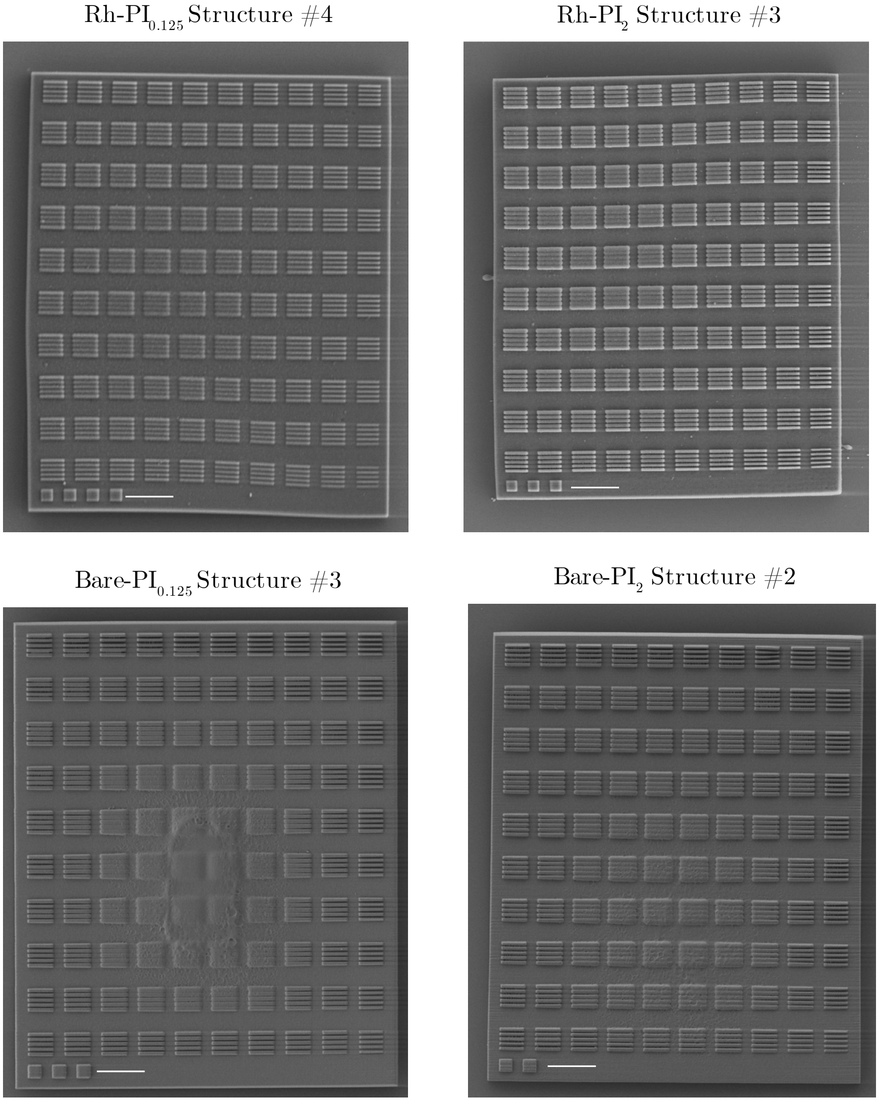

Figure S6. SEM micrographs of 2PP-printed Rh-PI$_{0.125}$, Bare-PI$_{0.125,}$ Rh-PI$_2$, and Bare-PI$_2$ samples at their optimal printing conditions found in submatrix structure #4, #3, #3, and #2, respectively. All the scale bars are 20 μm.

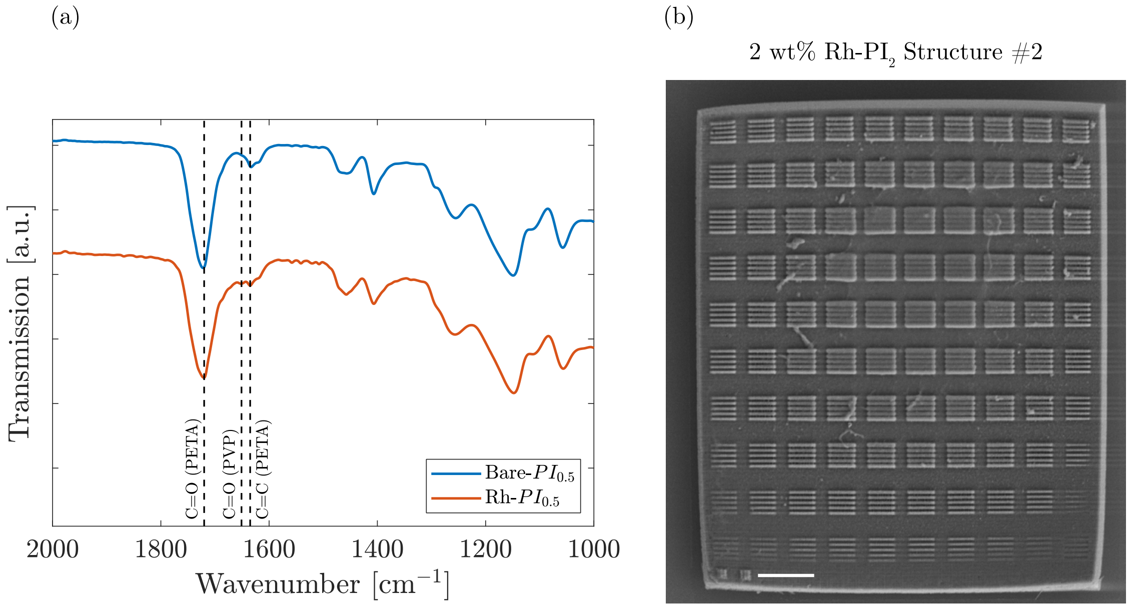

Figure S7. (a) FT-IR spectra of UV-polymerized Bare-and Rh-$PI_{0.5}$ films; the gray line represents the C=O band arising from the PVP on the Rh NP surface. (b) SEM micrograph of 2PP-printed structure with 2 wt.% Rh loading on the Rh-PI_2 resist formulation at optimal printing condition in submatrix structure #2. The scale bar is 20 µm.
